# Supplementary material for: Are carbon emissions trading and green financial instruments synergistic? -Comprehensive quantitative research based on content analysis
Source: PLoS One. 2024 Mar 7;19(3):e0298601. doi: 10.1371/journal.pone.0298601 (PMC10919601; doi:10.1371/journal.pone.0298601)
Supplement: S1 Data — (ZIP) [file pone.0298601.s001.zip › Data/地级市Z_F工作报告.zip!/─■╧─/╬Γ╓╥╩╨/└┤╫╘╣1⁄2╓┌║┼í╛┬φ┐╦╩2╛▌═°í┐_2014.docx]

各位代表：

现在，我代表市人民政府向大会报告政府工作，请予审议，并请市政协委员和其他列席人员提出意见。

2013年工作回顾

2013年是本届政府的开局之年。一年来，在自治区党委、政府和市委的正确领导下，在市人大、政协的有力支持下，全市上下攻坚克难，全力做好保增长、转方式、惠民生、促和谐各项工作，较好地完成了市四届人大一次会议确定的各项目标任务。预计全年完成地区生产总值350亿元，增长12%。公共财政预算收入31亿元，可比增长21%。完成固定资产投资495亿元，增长30%。实现社会消费品零售总额78.7亿元，增长15%。城镇居民人均可支配收入19900元，农民人均纯收入7200元，分别增长12%。 一年来，我们主要做了以下工作：

（一）积极应对经济下行压力，着力抓项目、增投资、稳增长。年初，面对主要经济指标较大回落的严峻挑战，我们认真研判，科学调度，有效应对：一是切实发挥政策保障效应。相继出台了加快非公经济发展、金融支持实体经济、优化发展环境、重点项目帮扶机制等政策措施，有力促进了经济平稳较快发展。二是加大财税金融支持力度。综合运用财税、金融等手段，全力帮助企业克服困难。市县财政安排1.3亿元专项资金扶持工业发展，减免退税2.7亿元。中小微企业贷款余额139.7亿元，增长25.4%。共发放各类创业小额担保贷款8.5亿元。利用企业债券成功融资10亿元，着力化解保障性住房建设等资金难题。三是狠抓关键举措落实。越是困难，越是狠抓“两大任务”不放松。全年落实上争项目2373个，落实上争资金122亿元。引进实施招商引资项目485个，到位资金500亿元。强力推进省道101至罗山大道连接线、市医院迁建等一批基础设施、社会民生项目。行之有效的思路、政策，对症下药的措施、办法，扭转了不利局面，全市经济总体呈现“稳中有进、进中向好”的良好态势。

（二）坚定不移培育壮大产业，着力调结构、增效益、促转型。我们立足现有的产业发展基础，进一步完善产业规划，优化产业布局，做大产业规模。龙头引领、配套跟进、项目支撑、园区承载、产业集群的发展格局逐步显现。工业支撑作用进一步增强。实施2000万元以上的工业项目140个，万胜5万吨氨基酸、红双赢10万吨大米等44个项目建成投产。吴忠卷烟厂新增1万箱生产指标。新增规模以上企业113家，占全区新增规上企业35%。新增亿元企业10家。地方工业实现增加值62亿元，占全部规模以上工业的53%，增长16%，高于中央和区属企业9个百分点。工业园区完成基础设施投资10.3亿元，园区承载和聚集效应明显提升。预计全年规模以上工业实现增加值118亿元，增长15%。工业对经济增长的贡献率达到39.5 %，拉动经济增长5.3个百分点。现代农业进一步增效。粮食总产量达90万吨，实现“十连丰”。奶牛、肉牛、羊饲养量分别达到17.3万头、58万头、620万只。夏进、伊利、金宇等10个万头奶牛牧场加快建设，盐池万亩草畜产业示范区、红寺堡万亩葡萄基地、下马关万亩枸杞基地全面建成。种植优质水稻20万亩，“塞外香”成为中国驰名商标。新增设施农业1万亩，累计达到17.44万亩。市级以上农业产业化龙头企业达到154家，累计发展农民专业合作社1035家，成立家庭农场160家，流转土地72万亩。预计全年完成农业总产值97亿元，增长5%。第三产业进一步活跃。市区“十大市场”、“四大滨河商业广场”基本建成，北方农资城、恒美家居等7个市场投入运营。深入开展特色餐饮业品牌提升活动。加快沿河文化旅游产业带建设，黄河大峡谷·中华黄河坛被评定为国家4A级景区。预计全年接待游客222万人次，实现旅游收入12.88亿元。完成房地产开发投资43.32亿元，增长88%，全市销售商品房面积114.32万平方米，增长48%。

（三）扎实推进城乡一体化发展，着力夯基础、增内涵、 提品位。开工城乡建设项目234个，完成投资189亿元，全市城市化率达到51%，市区城市化率达到58%。文体会展中心、秦渠文化休闲公园、乃光湖公园、黄河外滩大饭店等项目顺利推进。完成70万平米老旧小区节能改造，打通拓宽44条断头路。实施城市供排水、供热、亮化等一批市政基础设施建设，城市功能进一步完善。红寺堡生态公园、博大商业广场建成。盐池、同心旧城改造及新区开发等大县城建设重点项目深入推进。新建“幸福村庄”示范点24个，改造危房8684户。新建农村公路1000公里。牛首山“三纵六横”规划区新增公路190公里。主干道路大整治大绿化工作取得初步成果。创建“国家智慧城市”、“全国建筑节能示范城市”工作扎实推进。

（四）全面抓好事关全局的战略重点，着力挖潜力、增动力、强活力。我们充分挖掘自身优势，利用各方条件，在事关全局性的重点领域实现新的作为。慈善产业稳步提升。弘德包装40万箱烟包、中石化援建7500吨塑料制品等12个项目建成投产，解决就业岗位4000多个。慈善园区实现工业产值36亿元。建成慈善超市42家、慈善文化中心1家。承办“5·12黄河善谷行”慈善活动，慈善吴忠的对外影响力和知名度进一步扩大。生态环境持续向好。大力实施生态立市战略，完成营造林45.5万亩，森林面积达到420万亩，森林覆盖率13.5%。城市建成区绿地率、绿化覆盖率、人均公园绿地面积分别达到31.5%、37.3%和16.7平方米。我市荣获“全国绿化模范城市”殊荣，被环保部列为“全国生态文明建设试点地区”。加快推进国家环保模范城市创建工作，万元GDP能耗同比下降9.3%，提前三年完成自治区下达的“十二五”节能降耗目标。全年空气质量二级以上优良天数达到335天，居全区首位。对外开放取得实效。与宁波建立友好合作城市关系。与中国自动化集团达成建设自动化产业基地框架协议，与马来西亚狄维娜食品集团签订合作备忘录。

（五）把保障和改善民生放在更加突出位置，着力谋民利、惠民生、增福祉。全市公共财政投入民生和社会事业领域的资金达到123亿元，占公共财政预算支出的84.2%。市财政投入1.3亿元，为民办理的15件实事全面完成。新建、续建移民安置区7个，建成移民住房5044套，搬迁安置移民4463户18823人。盐池县提前两年完成“十二五”移民搬迁任务。深化全民创业，创办小企业750 个、创造新岗位 7400个、城镇新增就业 1.5万人、农村劳动力转移就业 24万人，实现劳务工资性收入18.7亿元。城镇登记失业率控制在4％以内。城乡居民基本医疗保险、养老保险参保率分别达到98%和86.6%。新建、续建139万平米保障性住房。市财政拿出937万元，为7.8万名60岁以上城乡居民每人每月增加10元基础养老金。为5192名残疾人发放居家托养补助363万元，实现全覆盖。全市安排3811万元，走访慰问困难群众。为12457名贫困家庭学生发放助学资金2086万元，没让一个学生因家庭困难而放弃学业，误了前程。投资5000万元，新购公交车71辆，新增城市公交线路5条，进一步方便群众出行。坚持教育优先发展战略，新建、改建学校150所、幼儿园27所，投资7317万元配置教育教学仪器设备，利通区、青铜峡市顺利通过自治区义务教育均衡发展验收。医药卫生体制改革深入推进，基本药物制度实现全覆盖。市人民医院迁建工程进展顺利，新建152个标准化村卫生室，基层医疗卫生服务条件得到明显改善。人口出生率11.76‰，出生政策符合率91.04%。文化体育事业繁荣发展，歌舞剧《曼苏尔》获第三届中国少数民族戏剧会演综合表演银奖。市文化艺术馆和博物馆完成布展工作，正式对外开放。成功举办第四届黄河金岸国际马拉松赛、全国青少年航空航天模型锦标赛和市第三届运动会。国防动员、双拥、科技、档案、统计、妇女儿童、住房公积金等工作取得新成效。

（六）狠抓依法行政和效能提升，着力转作风、促和谐、 保稳定。修订完善《市政府工作规则》，建立健全财政资金使用、国有资产管理、政府投资项目决算审计等多项制度，政府运行更加规范高效。深入推进依法行政，切实提升行政效能。取消、合并、下放30项行政许可事项，压缩法定办理时限2580个工作日，压缩幅度达到43%。建成统一高效的公共资源交易中心，工程招投标、政府采购等交易活动更加规范。坚决执行中央、自治区关于改进作风、密切联系群众的各项规定，工作作风持续好转，发展环境进一步优化。在全区群众评议机关和干部作风活动中，我市由上年的第四名进位到第二名。深入开展矛盾纠纷排查调处工作，严厉打击各类违法犯罪，八类案件破案率位居全区第一。全面启动新一轮禁毒人民战争。安全生产、应急管理和防震减灾工作不断加强。依法管理宗教事务，加大对宗教领域矛盾纠纷调处力度。我市被国家民委列为创建“全国民族团结进步示范市”首批试点。高度重视并自觉接受人大法律监督和政协民主监督。办理人大代表议案建议78件，政协委员提案255件，办复率均达100%。支持工会、共青团、妇联等人民团体积极开展工作。

各位代表，一年的发展实践使我们深切地体会到：之所以能够在挑战中逆势突破、在困难中奋力前行，得益于市委的坚强领导、人大政协的有力支持；得益于各级班子的精诚团结、各级干部的真抓实干；得益于全市上下的携手奋进、各族群众的拥护支持；得益于风清气正的良好环境、政通人和的大好局面。在此，我代表市人民政府，向全市广大工人、农民、知识分子、干部职工，向人大代表、政协委员、民主党派、工商联、人民团体、离退休老同志和社会各界人士，向驻吴人民解放军、武警和消防官兵、政法干警，向中央及区属驻吴单位，表示崇高的敬意和诚挚的感谢！

在肯定成绩的同时，我们也清醒地看到，经济社会发展还存在许多困难和问题。主要是：经济总量仍然偏小，财政实力弱、收支矛盾突出。工业支撑作用不强，农业产业化水平低，服务业发展层次不高，产业结构有待进一步优化。城乡、山川发展不平衡，扶贫攻坚任务依然艰巨。保障和改善民生的任务较重，教育、医疗等民生事业与群众期望还有较大差距。发展环境还需进一步优化，社会治理有待进一步加强等。对于这些问题，我们必须高度重视，采取有力措施，切实加以解决，努力把各方面工作做得更好。

2014年工作安排

今年是全面贯彻落实党的十八届三中全会精神的开局之年，是我市的“改革创新年”，扎实做好今年各项工作，尤为重要。我们必须正确研判宏观形势的发展变化，牢牢掌握工作主动权。

首先，吴忠正处在爬坡追赶的关键时期，面临着增长与转型的双重压力，做大总量与转型发展同样紧迫。现阶段发展不足、粗放发展仍然是吴忠最大的市情，发展的基本模式仍然是投资拉动型、资源依赖型，发展的主要任务仍然是在稳增长中调结构、在调结构中促增长。因此，加快发展、做大总量、转型升级仍然是解决所有问题的关键。

其次，当前的宏观形势依然偏紧，但有利因素也在增多，困难挑战与有利条件交织并存。一方面，我国经济稳中回升的基础还不稳固，下行压力依然较大，各类结构性问题和深层次矛盾依然较多。另一方面，国家继续实施积极的财政政策和稳健的货币政策；支持东部劳动密集型产业优先向中西部地区转移，中央财政转移支付将更多向中西部尤其是贫困地区倾斜；通过培育地方主体税种增加地方政府收入等。这些都将为我们加快发展带来重大利好。

第三，未来的改革力度将进一步加大，红利将不断释放，改革机遇与自身优势叠加发力。从全局看，十八届三中全会对全面深化改革作出总体部署。随着改革的纵深推进，各项改革措施的效应将持续显现，经济增长的动力和活力将不断增强。从自身看，吴忠是沿黄经济带的核心区，大银川都市区的重要组成部分。装备制造、能源化工等传统产业正在转型升级，一批成长壮大起来的大中型企业将成为新的经济增长点。吴忠进入了自身优势释放和改革机遇叠加的黄金发展期。

总的来看，尽管当前宏观经济环境仍存在一些不确定因素，我们发展过程中还面临着各种困难和问题，但只要我们抓紧抓牢大好机遇，充分利用各种有利条件，吴忠的发展潜力巨大，前景广阔，充满着无限希望。

根据中央、自治区、市委的一系列决策部署，今年政府工作的总体要求是：全面贯彻落实党的十八大、十八届三中全会精神，把改革创新贯穿于经济社会发展各个领域各个环节，坚持稳中求进、进中求好、好中求快，把壮大产业、转型升级作为主攻方向，把项目建设作为第一引擎，加快新型城镇化进程，加快生态文明建设，加快民生改善，全面建设开放吴忠、富裕吴忠、和谐吴忠、美丽吴忠、智慧吴忠，为完成“十二五”规划目标、全面建成小康社会打下更加坚实的基础。

全市经济社会发展主要预期目标和约束性指标是：地区生产总值增长11%左右；地方公共财政预算收入增长15%；社会消费品零售总额增长13%；全社会固定资产投资增长25%；城镇居民人均可支配收入增长11%，农民人均纯收入增长12%；城镇登记失业率控制在4.5%以内；居民消费价格涨幅控制在3.5%以内。完成自治区下达的节能减排目标。

各位代表，实现上述目标，我们必须以市委四届五次全体（扩大）会议确定的“改革开放、转型发展” 作为各项工作的主基调，以改革统领全局，用改革的精神、思路和办法破解发展难题、激发活力、推动发展。必须把扩量增效作为改革开放、转型发展的首要任务。进一步加压鼓劲，奋力追赶，使经济保持尽可能快的增长速度。在做大总量的同时，更加注重结构、质量和效益，推动经济转型升级。必须把“两大任务”作为改革开放、转型发展的主要抓手。坚持对内抓项目、对外引项目、对上争项目，确保项目质量优化提升。想方设法扩大政府投资、千方百计激活社会投资、竭尽全力引进市外投资，确保投资总量持续增长。必须把 创新驱动作为改革开放、转型发展的根本动力。改革是今年工作的主旋律，优化发展环境是关键。我们将在行政审批、行政收费、非公经济发展、财税金融改革、农村土地制度改革等方面，采取更具突破性举措，形成更富活力的体制机制。必须把生态建设 作为改革开放、转型发展的重要前提。优美的环境是我市最大的优势，一定要倍加珍惜，不断丰富和提升生态文明建设内涵和水平，使天更蓝、水更清、山更绿、空气更清新。必须把改善民生作为改革开放、转型发展的根本目的。把人民对美好生活的向往作为最高奋斗目标，切实解决群众上学、就业、医疗、住房、安全等方面的困难和问题，努力让全市人民生活的更富有、更自豪、更幸福。

重点做好以下九个方面的工作：

（一）坚持项目带动，在投资拉动经济增长上取得新进展。坚定不移的实施“两大任务”不动摇，确保上争项目资金、招商引资到位资金不低于上年。在加快项目建设上下功夫。实施农林水牧、工业、交通、社会事业等领域803个项目，完成年度投资618亿元。重点抓好自动化仪表生产基地、吴忠热电厂、伊利二期15万吨液态奶等产业项目；黄河吴忠市区段综合治理续建、101省道至罗山大道连接线、供热集污管网建设等基础设施项目；文体会展中心、义务教育均衡发展、保障性住房、城乡统筹等社会民生项目，尽快形成投资拉动效应。在加强项目前期工作上下功夫。紧盯国家、自治区投资方向和重点，全力以赴争取支持，力争一批基础设施、生态环境、民生事业等方面的项目列入国家和自治区投资计划。大力开展以商招商和产业链招商。紧盯新兴际华、中煤集团、北汽福田等大企业，争取引进2-3家投资过10亿元的大项目。进一步完善招商引资目标管理及考核办法，突出重点、注重实效、强化落地。在优化项目服务机制上下功夫。继续实施市级领导包抓项目责任制，严格落实项目督查和考核制度。制定项目推进路线图、时间表，紧盯进度、严把质量。各项工作围绕项目转，各方面力量围绕项目使，切实解决好规划、征地、资金等问题，为项目建设提供优质高效服务。

（二）立足转型升级，在加快新型工业化发展上再造新优势。进一步做大增量、调优存量，实施5000万元以上重点工业项目100个，确保规模以上工业增加值增长13%以上。做大做强食品产业。加快宝迪、伊佳、金麦尔等项目建设进度，进一步扩张规模、提升层次、培育品牌，让大企业顶天立地、让小企业铺天盖地。改造提升传统产业。鼓励支持冶金、建材等传统工业运用新工艺、新技术、新设备，提高科技水平，加快转型升级。着力提升以庆华煤化、金裕海石化等为龙头的能源化工业；以吴忠仪表、共创光伏等为龙头的装备制造业；以青铜峡铝业、太阳镁业等为龙头的新材料产业。扶优扶强中小企业。鼓励本土企业通过增资扩能、战略合作等途径，扩大规模，增强实力。积极扶持中小微企业发展，重点解决配套、融资、用工等难题。鼓励大企业通过专业分工、订单生产等方式，优先和市域内中小企业开展协作，加速构建上下游产品有序链接，大中小企业融合配套的产业发展新格局。力争年内新增规模以上企业20家、亿元企业10家、自治区级“两化融合”试点企业10家。提升园区发展水平。根据各工业园区产业基础和资源禀赋，进一步调整、优化工业园区功能定位和产业布局，引导园区从规模扩张向产业集聚转变、从政策优惠向环境优化转变。积极协调建立与银川综合保税区联动发展机制，使园区企业能享受保税区相关政策。探索推进与宁波等发达地区联合办园模式，吸收产业、人才、资金、管理优势，实现双赢。

（三）注重提质增效，在提高现代农业发展水平上迈出新步伐。以农民增收为核心，大力发展生态农业、节水农业，走特色、高品质、高效益、高端市场的“一特三高”发展路子，力争农业总产值增长5%以上。扩大规模，打造特色产业带。以保障粮食安全为基础，全市优质粮食种植面积稳定在100万亩，粮食产量达到90万吨。加快四条农业示范带建设，坚持“一县一业”，重点在奶产业、牛羊肉、优质大米、酿酒葡萄、有机枸杞上扩大规模、提升品质。新增奶牛3万头、肉牛5万头、滩羊20万只、酿酒葡萄2万亩、枸杞中药材3万亩。以果蔬为主的设施农业稳定在17万亩以上。严格落实“菜篮子”责任制，每个县（市、区）都要建设一定规模的永久性蔬菜基地。加快农业龙头企业生产基地建设。整合改造老旧养殖园区，发挥存量资源效益。提高吴忠国家农业科技园区建设水平，打造生态农业“有机谷”。壮大龙头，提升市场带动力。大力扶持龙头企业，加快培育农民专业合作社、家庭农场等新型经营主体。鼓励和支持土地向专业大户、家庭农场、农民专业合作社流转，年内培育市级以上农民专业合作社20家，家庭农场100家，流转土地面积100万亩。加快推进特色产业标准化、品牌化、市场化生产，完善农产品质量源头控制、检验监测、认证等保障服务体系。重点支持伊利乳业、塞外香大米、易捷枸杞等农业龙头企业扩规模、拓市场，培育一批宁夏知名、叫响全国的农字号品牌。夯实基础，提高综合生产力。因地制宜开展农田水利基本建设，实施苦水河治理、小农水重点县、安全饮水等一批水利工程，新增节水灌溉25万亩，治理水土流失240平方公里，解决2万人安全饮水问题，实现农村安全饮水工程全覆盖。推广农业科技新品种、新技术、新装备、新成果，提升农业科技水平。

（四）加快融合互动，在激发服务业发展活力上拓展新途径。充分聚合优势资源，大力推进商贸物流、特色餐饮、金融保险、电子商务、文化旅游等业态融合发展。加快传统商贸业改造。推进“十大市场”和县级专业市场建设与运营，引进大型物流企业，逐步形成区域性物流集散地。加快特色餐饮业提档。深入开展特色餐饮业品牌提升活动，鼓励和支持餐饮名店发展连锁经营。培育1-2家营业额过5000万元的餐饮集团，10家营业额在1000万元以上的餐饮企业，建成具有一定规模和影响力的餐饮特色名镇、名街、名店、名农家。加快文化旅游业融合。重点发展以黄河文化游、红色经典游、绿色生态游和近郊休闲游为主的特色旅游经济。加快完善黄河楼、108塔、大禹文化园等基础设施建设，着力打造沿河文化旅游产业带，加快构建黄河文化旅游精品线路。整合资源，组建文化旅游发展有限公司，推进全市文化旅游资源集约化、规模化经营。力争接待游客和旅游收入分别增长12%和15%。加快现代服务业培育。大力发展电子商务、邮政快递、设计研发、咨询中介、休闲娱乐、体育健身、养老家政等新兴消费业态。争创国家电子商务示范城市。

（五）着眼城乡统筹，在推进城乡一体化发展上谱写新篇章。全年实施城乡建设项目170个，完成投资180亿元。全市城市化率提高2个百分点。坚持内涵发展，做美黄河金岸。大力实施 “西移东扩、南控北拓”战略。加快利青一体化步伐，实施文化体育会展中心、黄河观光游览、秦渠综合整治二期、乃光湖公园二期等40个重点项目。新建、续建南二环等14条城市道路和15公里排水工程，改造老城区20公里供热管网。新建、续建恒昌幸福城等100万平米房地产开发项目。积极主动融入大银川都市区建设，推进银吴同城，吴灵（武）相交、吴永（宁）对接，形成优势、产业、功能互补，竞相发展的新格局。坚持产城融合，打造活力县城。引导支持各县（市、区）从资源禀赋和产业基础出发，培育壮大优势主导产业，逐步打造特色鲜明、错位发展的县域经济方阵。加快县城基础设施建设，完善公共服务配套，不断改善居住条件，进一步提升大县城集聚承载力和辐射带动力。坚持人文一体，建设优美乡村。加大村镇整合聚集力度，提升金银滩、峡口、下马关、柳泉、惠安堡等特色城镇建设水平。努力培育一批功能完备、特色彰显、环境优美、宜居宜业的新城镇。新建、续建30个“美丽村庄”示范点，改造农村危房5000户。加大农村公共基础设施建设力度，实现乡村道路硬化、卫生净化、村庄绿化、环境美化。坚持建管一体，打造宜居城市。坚持精品化建设、精细化管理城市。加快旧城改造步伐，加强物业管理，不断完善小街小巷、老旧小区、安置小区等基础设施。合理增加停车泊位，优化公交线路布局。深入实施城市净化、美化、绿化、亮化工程，使市容更整洁、出行更顺畅、景观更宜人、城市更宜居，全面提高城市管理水平。坚持资源整合，打造智慧城市。加快推进智慧规划、智慧服务、智慧管理等重点工程建设，建成智慧吴忠数字资源库。逐步构筑贯穿城市生产生活、安全管理、公共服务、社会运行等智慧应用体系，促进信息化与经济社会发展深度融合，让老百姓在智慧城市里生活的更美好。

（六）建设美丽吴忠，在加强生态文明建设上展示新形象。加快创建国家环保模范城市，扎实开展全国生态文明建设试点工作，进一步彰显吴忠的水韵之美、自然之美、绿色之美。全方位推进生态建设。全市营造林30万亩，森林面积达到435万亩，森林覆盖率达到14%。新增城市绿化5000亩，完成主干道路绿化美化6.95万亩。启动“黄河善谷”植物园建设。继续实施退耕还林、退牧还草和三北防护林、防沙治沙、天然林保护工程。大力度 发展循环经济。推广应用清洁能源，加大节能降耗力度，坚决淘汰落后产能。重点实施15项工业节能技改和循环经济项目。严把新上项目节能评估关、环保准入关。严格环保执法，加强对重点监控区域和重点企业的环境监管，确保污染物达标排放。多举措加强综合整治。加强森林、水系、湿地及水源涵养区、生态脆弱区的环境保护。综合防治化工污染、重金属污染和农业面源污染。扎实推进生态县、生态乡村和环境友好型社区等创建活动。健全生态环境保护责任追究制度和环境损害赔偿制度，让保护者获得收益，让污染者付出代价。开展空气质量PM2.5监测，确保空气质量二级以上优良天数达到90%以上。

（七）强化创新驱动，在深化关键领域改革上实现新突破。面对新形势新任务，必须坚定不移地解放思想，大力推进改革创新，向改革要动力、要活力、要红利。以政务服务创新推动发展环境优化。认真落实地方政府机构改革中各项工作任务。加快行政审批制度改革，实行“两个清单、三个一律”，力争全市行政审批事项减少60%以上，平均办结时限压缩60%。推行“项目四级服务制”，最大限度为企业、法人、公民创业提供方便。率先在服务行业实施“先业次照再证后规范”的工商注册程序。在通过安全、环保评价的基础上，降低企业准入门槛，减少项目审批环节，取消市级收费，涉及国家和自治区的收费项目一律由财政埋单。以财税金融改革创新推动融资总量突破。完善公共财政体系，加大财源建设力度，加强税收征管，优化财政支出结构，提高财政资金使用效益。建立财政风险监控和预警机制，把政府负债控制在合理区间，防止过度举债搞建设。支持金融业务创新。积极引进各类金融机构在我市设立网点、开办业务。鼓励发展民营金融机构。吸纳社会资本增资扩股，做大做强市本级担保机构，着力提升融资担保能力。支持企业债券和中期票据发行，继续做好区域集优债发行工作，选择3家企业进入“新三板”市场交易。以用地制度创新推动土地难题破解。鼓励农村承包地自愿有序流转、抵押、租赁。在利通区、青铜峡市开展城市规划区内农民宅基地换房试点，规划区外的宅基地可在市公共资源交易中心交易。每个县（市、区）选择1-2个乡镇开展农村集体资产股份改革试点。以对外开放创新推动发展活力增强。探索市场化运作模式，在促进招商引资、展示城市魅力、扩大对外宣传等方面取得实效。充分利用地缘优势、人文优势，找准突破口，制定丝绸之路经济带行动计划。大力发展慈善产业，完善政策措施，建设慈善城市，吸引中外爱心人士来吴投资置业兴善。

（八）突出民生优先，在保障群众切身利益上争取新作为。按照保基本、兜底线、促公平、可持续的原则，促进各项社会事业协调发展，进一步提高社会保障水平。年内高质量办好为民承诺的实事。扎实推进生态移民和扶贫攻坚。新建生态移民住房1959套，搬迁安置移民3873户1.6万人，全面完成“十二五”生态移民建设任务。加快完善移民村水电路、学校、医务室、村级活动中心等配套基础设施。统筹做好产业发展、就业创业、社会治理等工作，确保移民群众安置有着落、发展有基础、生活有保障、致富有渠道。加强资源整合，加大100个贫困村整村推进力度。统筹社会扶贫力量，群众不脱贫、单位不脱钩。不断提高社会保障能力。深化全民创业就业，加大对高校毕业生、失地农民等群体就业援助和技能培训力度，推动更高质量就业，力争全年创办小企业650个，创造新岗位6500个。推进社会保险提标扩面，医疗保险、养老保险参保率分别达到98%、90%以上，并稳步提高保障水平。启动实施城乡居民大病医疗保险，让重病百姓不硬扛、有希望。继续提高社会保障和救助标准，让困难群众有念想。建设保障性住房8000套，棚户区改造10000套，让低收入家庭有指望。鼓励社会力量以多种形式发展养老事业，让老人、家庭、社会无后忧。着力促进教育均衡发展。加大教育投入，改善办学条件，进一步缩小校际差距。减轻学生课业负担，缓解大班额问题，让孩子平等、快乐地接受教育。实行教师县级统一管理，建立政策导向机制，使校长和教师轮岗交流常态化。年内新建、改建城乡学校25所，幼儿园27所，建设标准化学校328所，确保利通区、青铜峡市顺利通过国家义务教育均衡发展评估验收。提高普通高中办学水平，加快现代职业教育体系建设，稳步提升教育教学质量。大力促进医疗卫生事业发展。加快市人民医院迁建工程。办好县级人民医院，推进社区、村卫生室标准化建设，提升基层医疗卫生服务能力，方便群众就医。支持非公立医疗机构规范发展，促进回医回药健康发展。落实新的生育政策，强化优生优育，稳定低生育水平。加快推进文化事业发展。积极构建覆盖城乡、惠及全民的公共文化服务体系。组织开展送戏下乡、广场文化、全民健身等文化体育活动。鼓励文艺精品创作。年内完成董府修缮改造工程，向公众开放。

（九）围绕和谐稳定，在加强和创新社会治理上开创新局面。着力提升社会治理水平，努力实现政府治理、社会自我调节和居民自治良性互动。加强民族宗教工作。以创建“全国民族团结进步模范市”为契机，扎实开展“民族团结进步创建攻坚年”活动，进一步巩固提升创建成果。依法加强和创新宗教事务管理，着力提高管理服务水平。及时化解宗教领域矛盾纠纷，坚决打击非法宗教活动，巩固民族团结、宗教和顺、社会稳定的良好局面。 加强社会综合治理。建立畅通有序的诉求表达、心理干预、矛盾调处、权益保障机制，及时化解社会矛盾，努力实现案结、事了、人和。深化社区管理创新，增强城乡社区服务功能和自治能力。加强社会舆情信息管控，强化对社会热点问题的正面引导。严格落实安全生产责任，突出抓好煤炭、化工、食品药品、公共场所等重点领域安全生产监管，坚决遏制重特大事故发生。完善应急管理体制机制，提升突发事件应对和处置工作水平。全面打好新一轮禁毒人民战争，依法严厉打击各类犯罪活动，让群众生活更安宁。提升社会文明程度。加强社会主义核心价值体系建设，用“中国梦”、“五个吴忠”的宏伟目标凝聚人心。深入推进文明城市创建，进一步增强城市凝聚力和市民归属感。广泛开展以立德树人、争做“美丽吴忠人”为主题的精神文明创建活动，使全市人民精神风貌更加昂扬向上。

进一步加强统计工作，完成第三次全国经济普查任务。积极促进军地融合发展，抓好国防教育、国防动员和民兵预备役工作，提高双拥共建水平。加强妇女儿童、外事侨务、气象、档案、市志、防震、住房公积金等工作。支持工会、共青团、妇联等人民团体工作。

切实加强政府自身建设

新形势、新目标、新任务对政府工作提出了更高要求，我们必须以改革创新的精神努力建设为民、务实、高效、廉洁政府，受人民监督、为人民服务，让人民满意。

（一）建设责任政府。面对新要求、新矛盾、新问题，用学习、境界、创新敢于担当，以定力、心力、能力勇于负责，踏石留印、抓铁有痕、奋发有为。盯住不落实的事，严查不落实的人，确保政府部门和各级干部认真履行职责，促进重点工作、重点项目件件落到实处。

（二）建设法治政府。坚持把依法行政贯穿政府工作各个领域、各个环节。加强行政执法监督，切实做到公正文明执法。 自觉接受人大法律监督和政协民主监督，高度重视司法监督、舆论监督和社会公众监督。认真听取民主党派、工商联、无党派人士和人民团体的意见建议。健全完善重大行政决策公众参与、专家论证、风险评估等制度，提高政府决策的科学化、民主化、法制化水平。

（三）建设服务政府。始终把全心全意为人民服务作为政府工作的根本宗旨。深入开展以为民务实清廉为主要内容的群众路线教育实践活动，时刻把群众的安危冷暖放在心上。集中精力抓好经济发展、市场监管、公共服务、社会治理和生态保护，着力打造“两优”、“五地”环境。加强政府诚信建设，坚持诚信办事、诚信服务，树立有诺必践的政府形象。以我们的实际行动让全社会感受到市场环境、创业条件、干部作风在一天天好转。

（四）建设阳光政府。始终把廉洁从政作为政府工作的底线。牢固树立长期过紧日子思想，严格控制“三公”经费，真正把有限的财力用在最急需的地方、用在改善民生最迫切的事情上。坚持用制度管权、管事、管人。强化重点领域、关键环节的行政监察，加强财政资金、重大项目和民生工程的审计监督。严肃查处违纪违法行为，坚决纠正损害群众利益的突出问题。加快政府决策行为、活动信息及财政预算公开，让群众知道政府花了多少钱，办了什么事。让百姓看得到、看得懂、看得明白。

各位代表，吴忠的发展充满希望，吴忠的前景无限美好！让我们高举中国特色社会主义伟大旗帜，在自治区党委、政府和市委的坚强领导下，凝心聚力，开拓进取，为建设开放、富裕、和谐、美丽、智慧新吴忠，建成全面小康社会而努力奋斗！
